# Genome-Wide Identification and Expression Profiling of Tomato *Hsp20* Gene Family in Response to Biotic and Abiotic Stresses

**DOI:** 10.3389/fpls.2016.01215

**Published:** 2016-08-17

**Authors:** Jiahong Yu, Yuan Cheng, Kun Feng, Meiying Ruan, Qingjing Ye, Rongqing Wang, Zhimiao Li, Guozhi Zhou, Zhuping Yao, Yuejian Yang, Hongjian Wan

**Affiliations:** ^1^State Key Laboratory Breeding Base for Zhejiang Sustainable Pest and Disease Control, Institute of Vegetables, Zhejiang Academy of Agricultural SciencesHangzhou, China; ^2^College of Chemistry and Life Science, Zhejiang Normal UniversityJinhua, China

**Keywords:** heat shock protein 20, gene organization, phylogenetic relationship, expression profile, abiotic and biotic stresses

## Abstract

The Hsp20 genes are involved in the response of plants to environment stresses including heat shock and also play a vital role in plant growth and development. They represent the most abundant small heat shock proteins (sHsps) in plants, but little is known about this family in tomato (*Solanum lycopersicum*), an important vegetable crop in the world. Here, we characterized heat shock protein 20 (*SlHsp20*) gene family in tomato through integration of gene structure, chromosome location, phylogenetic relationship, and expression profile. Using bioinformatics-based methods, we identified at least 42 putative *SlHsp20* genes in tomato. Sequence analysis revealed that most of *SlHsp20* genes possessed no intron or a relatively short intron in length. Chromosome mapping indicated that inter-arm and intra-chromosome duplication events contributed remarkably to the expansion of *SlHsp20* genes. Phylogentic tree of *Hsp20* genes from tomato and other plant species revealed that *SlHsp20* genes were grouped into 13 subfamilies, indicating that these genes may have a common ancestor that generated diverse subfamilies prior to the mono-dicot split. In addition, expression analysis using RNA-seq in various tissues and developmental stages of cultivated tomato and the wild relative *Solanum pimpinellifolium* revealed that most of these genes (83%) were expressed in at least one stage from at least one genotype. Out of 42 genes, 4 genes were expressed constitutively in almost all the tissues analyzed, implying that these genes might have specific housekeeping function in tomato cell under normal growth conditions. Two *SlHsp20* genes displayed differential expression levels between cultivated tomato and *S. pimpinellifolium* in vegetative (leaf and root) and reproductive organs (floral bud and flower), suggesting inter-species diversification for functional specialization during the process of domestication. Based on genome-wide microarray analysis, we showed that the transcript levels of *SlHsp20* genes could be induced profusely by abiotic and biotic stresses such as heat, drought, salt, *Botrytis cinerea*, and Tomato Spotted Wilt Virus (TSWV), indicating their potential roles in mediating the response of tomato plants to environment stresses. In conclusion, these results provide valuable information for elucidating the evolutionary relationship of *Hsp20* gene family and functional characterization of the *SlHsp20* gene family in the future.

## Introduction

Plants live in a complex environment, where multiple biotic and abiotic stresses may seriously restrict their growth and development (Cramer et al., 2011). In the recent years, due to unprecedented global warming caused by various factors, high temperature has appeared as one of the most severe abiotic stresses around the world. To survive and acclimatize under the adverse environment conditions, plants have established self-defense mechanisms during the course of long-term evolution. Heat shock proteins (Hsps), acknowledged as evolutionarily conserved ubiquitous proteins in all organisms, were first discovered in *Drosophila melanogaster* in response to the elevated temperature stress (Ritossa, 1962; Neta-Sharir et al., 2005; Cashikar et al., 2006). Previous studies have shown that high temperature as well as other environmental cues (cold, salinity, drought, heavy metals, anoxia, pathogens, etc.) could induce the occurrence of Hsps (Lindquist and Craig, 1988; Wang et al., 2003). In addition, the Hsps were also found to be associated with plant growth and development, such as embryogenesis, seed germination, and fruit maturation (Neta-Sharir et al., 2005).

According to sequence homology and molecular weight, Hsps in eukaryotes can be grouped into six families such as Hsp110, Hsp90, Hsp70, Hsp60, small heat shock protein (sHsp) of 15–42 kDa (or Hsp20) and ubiquitin (Carper et al., 1987; Sarkar et al., 2009). Out of these six groups of Hsps, sHsps are the most primary and abundant proteins under thermal stimulation in many species (Vierling et al., 1989; Vierling, 1991). Notably, among eukaryotes, the higher plants possessed more quantities of *Hsp20s* (Vierling, 1991). As Hsp20 is encoded by a multigene family, it is considered as the most ample and complicated member in Hsps (Vierling, 1991). The characteristic feature of Hsp20 is the presence of a carboxyl-terminal conserved domain of 80–100 amino acid residues, which can be defined as the α-crystallin domain (ACD). This highly conserved ACD, which is flanked by a short carboxyl-terminal extension and a variable amino-terminal domain, is believed to comprise two hydrophobic β-sheet motifs that are separated by a hydrophilic α-helical region of variable length (de Jong et al., 1998). Moreover, the *Hsp20* gene family also exhibits extensive sequence variability and evolutionary divergence, which is remarkably different from other families of Hsps (Basha et al., 2012).

An earlier study showed that Hsp20 proteins in eukaryotes, which are collectively known as molecular chaperones, function as multimeric complexes ranging from 8 to 24 or more subunits (Van Montfort et al., 2002). These chaperons can selectively bind to partially folded or denatured proteins in an ATP-independent manner, which can prevent proteins from the irreversible aggregation and facilitate them folding properly (Lee and Vierling, 2000; Sun et al., 2002; Van Montfort et al., 2002). Recent studies revealed that these chaperones were important for disease resistance triggered by resistance (R) proteins and played a fundamental role in plant immunity (Botër et al., 2007; Shirasu, 2009).

To date, the *Hsp20* gene families have been investigated in several plant species, including *Arabidopsis*, rice, soybean, pepper, and *Populus trchocarpa* (Scharf et al., 2001; Waters et al., 2008; Ouyang et al., 2009; Sarkar et al., 2009; Lopes-Caitar et al., 2013; Guo et al., 2015). In addition, some key features of Hsp20 and biologic function of several *Hsp20* genes had been identified (Nautiyal and Shono, 2010; Goyal et al., 2012; Huther et al., 2013; Mahesh et al., 2013; Arce et al., 2015; Zhang et al., 2016). Although the availability of the tomato whole-genome sequence provides valuable resources for getting into an in-depth understanding of *Hsp20s* (Sato et al., 2012), little information is available on the integrated Hsp20 family at whole genomic level in tomato.

In the current paper, the members of *SlHsp20* gene family in tomato were identified using a bioinformatics method and characterized by integration of sequence features, chromosome location, phylogenetic relationship, evolutionary origin, and expression patterns. These results provide valuable information that can be implicated in elucidating the evolutionary relationship of *Hsp20* gene family in higher plants and functional characterization of the *SlHsp20* gene family in the future.

## Materials and methods

### Retrieval and identification of *Hsp20* genes in tomato

In this paper, the predicted *SlHsp20* genes were identified as follows: firstly, the tomato genome sequences were downloaded from the database Sol Genomics Network (SGN, Release 2. 5, http://solgenomics.net/) and used to set up a local database by the software “DNATOOLs.” Secondly, the Hidden Markov Model (HMM) profile of Hsp20 domain (PF00011) from PFam (http://pfam.sanger.ac.uk/) was employed to search against the local database using BlastP method (*e* < 1e-5). Furthermore, *Hsp20* candidates with incomplete Hsp20 domain might be missed using HMM profile. Name search using the word “hsp20” as a keyword also applied to retrieve in SGN database. The redundant sequences were manually removed. Finally, all these predicted genes were examined for the Hsp20 domain in SMART (http://smart.embl-heidelberg.de/smart/batch.pl), Pfam and InterProScan (http://www.ebi.ac.uk/interpro/), and those without the common Hsp20 domain were excluded.

### Sequence analysis and structural characterization

Information of candidate *SlHsp20* genes was obtained via searching the SGN database (http://solgenomics.net/search/locus), including chromosome locations, intron numbers, genomic sequences, coding sequences (CDS), and amino acid sequences. Intron-exon structure was determined by alignment of genome DNA and full-length cDNA sequence using Gene Structure Display Server 2.0 (http://gsds.cbi.pku.edu.cn/) (Hu et al., 2015). Molecular weight, theoretical isoelectric point (theoretical p*I*), and instability index (II; with the value >40 classified as unstable) of SlHsp20 proteins were analyzed by using ProtParam tool (http://web.expasy.org/protparam/).

The putative protein sequences were subjected to MEME program (http://meme-suite.org/tools/meme) to investigate conserved motifs with the following parameters: site distribution—any number of repetitions, number of motifs—10, the motif width between 6 and 200.

### Phylogenetic analysis

To illuminate evolutionary relationship of *Hsp20* gene family, the representative *Hsp20* genes from *Arabidopsis*, rice, soybean, bluebunch wheatgrass, barley, common wheat, Eurasian aspen, together with *SlHsp20* genes from tomato, were selected for constructing phylogenetic tree (Ouyang et al., 2009; Sarkar et al., 2009; Lopes-Caitar et al., 2013). Multiple sequence alignment of Hsp20 proteins was conducted using ClustalX 1.83. An un-rooted Neighbor-joining phylogenetic tree was constructed using MEGA 7.0 software with default settings (Kumar et al., 2016). The bootstrap test was performed by 1000 replications.

Four Online tools were employed to predict subcellular localization, including, Predotar (https://urgi.versailles.inra.fr/Tools/Predotar), Wolf Psort (http://www.genscript.com/psort/wolf_psort.html), TargetP (http://www.cbs.dtu.dk/services/TargetP/), and MultiLoc (http://abi.inf.uni-tuebingen.de/Services/MultiLoc2). The prediction of signal peptide and transmembrane domain was performed with SMART program (http://smart.embl-heidelberg.de/smart/batch.pl).

### Chromosome localization and gene duplication

Chromosome mapping of the candidate *SlHsp20* genes was viewed using the software MapDraw V2.1 (Liu and Meng, 2003). Tandem duplication and segmental duplication were also further investigated. The former was confirmed with the following criteria: (1) an array of two or more *SlHsp20* genes within a range of 100 kb distance; (2) the alignment had a coverage rate more than 70% of the longer gene; (3) and the identity of the aligned region was no less than 70% (Li et al., 2010; Huang et al., 2012; Wei et al., 2016). The latter was identified based on Plant Genome Duplication Database (PGDD, http://chibba.agtec.uga.edu/duplication/index/locus).

### Tissue-specific expression analysis

In this study, RNA-seq data from Tomato Functional Genomics Database (TFGD, http://ted.bti.cornell.edu/cgi-bin/TFGD/digital/home.cgi) were used to investigate expression patterns of putative *SlHsp20* genes in different tissues of cultivated tomato (*Solanum lycopersicum*) and the wild relative (*Solanum pimpinellifolium*). Different tissues in cultivated tomato included leaves, roots, flower buds, fully opened flowers, 1 cm, 2 cm, 3 cm, mature green, breaker, and breaker+10 fruits. In the wild species (*S. pimpinellifolium*), nine tissues and organs were selected for analysis, including leaves, whole root, hypocotyl, cotyledons, flower buds 10 days before anthesis or younger, flowers at anthesis, 10 days post anthesis (DPA) fruit, 20 DPA fruit and breaker stage ripening fruit. Digital gene expression analysis of the putative *SlHsp20* gene family was performed using software MultiExperiment Viewer (MeV) (Howe et al., 2010).

### Expression profile of *SlHsp20* genes under different stress conditions

To get insight into the expression profiles of the *SlHsp20* gene family under different environmental stresses, microarray analysis was performed. Whole genome microarray data for diverse environment stresses such as heat, drought, salt, *Botrytis cinerea*, and Tomato Spotted Wilt Virus (TSWV) was downloaded from database TFGD (http://ted.bti.cornell.edu/). The array platforms for microarray data included TOM2 oligo array and Affymetrix genome array. For TOM2 oligo array, the probe sets of the *SlHsp20* genes were identified through BlastN analysis in the database “TOM2 oligo sequences.” For Affymetrix genome array, Probe Match tool in NetAffx Analysis Center (http://www.affymetrix.com) was used to obtain probe sequences. Average value was considered for *SlHsp20* genes that had more than one probe set. The expression values of *SlHsp20* genes that were up- or down-regulated more than two-fold with *P* < 0.05 were considered as differently expressed.

## Results

### Identification of Hsp20 family members in tomato

Name search and HMM analysis showed a total of 42 candidate *SlHsp20* genes, four of which were identified to contain incomplete Hsp20 domains. For convenience, the *SlHsp20* genes were named according to their molecular weight in our study. Details on gene name, locus name, chromosome location, open reading frame (ORF) length, intron number, protein length, molecular weight, isoelectric point (p*I*), and instability index were listed in Table 1.

**Table 1:** **Features of ***SlHsp20*** genes in tomato**.

**Name**	**SGN locus**	**Chromosome location**	**ORF (bp)**	**Introns**	**Protein length**	**Molecular weight (kD)**	**Point**	**Index**	**Domain**
SlHsp11.9	Solyc00g053740	Chr0:13824977–13825669	324	3	107	11.9	6.25	25.61	
SlHsp25.7A	Solyc01g009200	Chr 1:3241560–3240198	699	1	232	25.7	5.86	44.14	Transmembrane domain
SlHsp23.8A	Solyc01g009220	Chr 1:3247795–3246119	642	1	213	23.8	9.48	36.33	Transmembrane domain
SlHsp17.3A	Solyc01g017030	Chr 1:23550205–23554512	462	4	153	17.3	8.99	39.67	
SlHsp14.5	Solyc01g017790	Chr 1:25432286–25427746	390	5	129	14.5	6.94	34.03	
SlHsp15.8	Solyc01g018070	Chr 1:27398962–27394648	429	4	142	15.8	5.1	29.41	Transmembrane domain
SlHsp49.3	Solyc01g096960	Chr 1:87963575–87961920	1277	1	441	49.3	8.73	35.27	Transmembrane domain
SlHsp39.4	Solyc01g096980	Chr 1:87968194–87971404	1047	2	348	39.4	9.11	53.1	
SlHsp21.6A	Solyc01g102960	Chr 1:91610881–91611760	570	0	189	21.6	7.89	53.22	Signal peptide
SlHsp15.7	Solyc02g080410	Chr 2:44640649–44639813	414	1	137	15.7	4.91	44.72	
SlHsp15.6	Solyc02g093600	Chr 2:54402405–54403349	411	1	136	15.6	7.65	42.53	
SlHsp26.2	Solyc03g082420	Chr 3:45899828–45898742	708	1	236	26.2	7.84	34.18	
SlHsp23.7	Solyc03g113180	Chr 3:63421266–63422260	630	1	209	23.7	4.96	60.11	
SlHsp21.5A	Solyc03g113930	Chr 3:63978540–63979106	567	0	188	21.5	6.93	50.33	Signal peptide
SlHsp16.1A	Solyc03g123540	Chr 3:70366718–70367347	435	1	144	16.1	8.4	62.05	
SlHsp16.1B	Solyc04g014480	Chr 4:4722700–4724263	438	1	145	16.1	6.97	49.27	
SlHsp37.0	Solyc04g071490	Chr 4:58477607–58478832	978	1	325	37.0	5.83	36.54	Transmembrane domain
SlHsp17.9	Solyc04g072250	Chr 4:59249315–59251089	492	1	163	17.9	5.47	40.33	
SlHsp25.7B	Solyc05g014280	Chr 5:8089580–8092146	666	2	221	25.7	9.31	45.74	
SlHsp17.7A	Solyc06g076520	Chr 6:47546790–47547254	627	0	154	17.7	5.84	51.74	
SlHsp17.6A	Solyc06g076540	Chr 6:47551057–47551521	465	0	154	17.6	5.82	47.21	
SlHsp17.6B	Solyc06g076560	Chr 6:47559714–47560178	465	0	154	17.6	5.84	50.91	
SlHsp17.6C	Solyc06g076570	Chr 6:47564101–47564565	465	0	154	17.6	5.57	46.42	
SlHsp9.1	Solyc07g045610	Chr 7:58755057–58754174	237	1	78	9.1	5.17	23.22	
SlHsp26.5	Solyc07g055720	Chr 7:63655500–63657426	717	5	238	26.5	9.42	60.7	
SlHsp21.6B	Solyc07g064020	Chr 7:66320971–66322805	567	1	188	21.6	5.64	37.37	
SlHsp17.3B	Solyc08g062340	Chr 8:50913795–50913023	468	0	155	17.3	6.75	35.61	
SlHsp17.6D	Solyc08g062450	Chr 8:51109016–51109492	477	0	158	17.6	6.32	36.62	
SlHsp23.8B	Solyc08g078700	Chr 8:62469844–62471072	633	1	210	23.8	6.45	60.54	
SlHsp21.5B	Solyc08g078710	Chr 8:62472773–62473878	591	1	196	21.5	8.37	55.57	
SlHsp18.2	Solyc08g078720	Chr 8:62475339–62476959	507	1	168	18.2	5.06	34.01	
SlHsp26.8	Solyc09g007140	Chr 9:769674–771056	711	1	236	26.8	5.23	47.91	Transmembrane domain
SlHsp24.5	Solyc09g011710	Chr 9:4976527–4978000	627	1	208	24.5	7.15	59.67	
SlHsp15.2	Solyc09g015000	Chr 9:7427223–7428264	405	1	134	15.2	8.86	57.81	
SlHsp17.7B	Solyc09g015020	Chr 9:7440133–7440597	465	0	154	17.7	5.84	55.8	
SlHsp7.8	Solyc09g059210	Chr 9:53755532–53755735	204	0	67	7.8	4.69	48.31	
SlHsp15.5	Solyc10g076880	Chr 10:59862547–59863282	420	1	139	15.5	9.21	11.34	
SlHsp27.1	Solyc10g086680	Chr 10:65453568–65452864	705	0	234	27.1	9.48	37.94	
SlHsp21.5C	Solyc11g020330	Chr 11:10856316–10856888	573	0	190	21.5	5.75	39.6	Signal peptide
SlHsp27.5	Solyc11g071560	Chr 11:54984205–54985643	744	1	247	27.5	6.04	44.67	Transmembrane domain
SlHsp9.0	Solyc12g042830	Chr 12:39616918–39617157	240	0	79	9.0	4.53	38.02	
SlHsp27.2	Solyc12g056560	Chr 12:62506816–62507722	723	1	240	27.2	8.71	37.27	Transmembrane domain

The four *SlHsp20* genes with incomplete Hsp20 domain encoded truncated proteins (67–129 aa) and could be non- functional or pseudogenes. Therefore, these *SlHsp20* genes were excluded in phylogenetic tree construction. Molecular weight of the remaining predicted *SlHsp20* genes ranged from 15.2 to 49.3 kDa, except for *SlHsp11.9* and *SlHsp14.5* that were less than 15 kDa. Molecular weights of these SlHsp20 proteins had a large variation. Isoelectric points ranged from 4.53 (*SlHsp9.0*) to 9.8 (*SlHsp23.8A*), and protein length ranged from 67 (*SlHsp7.8*) to 441 aa (*SlHsp49.3*). The instability index indicates that 18 of the predicted SlHsp20 proteins were deemed to be stable proteins, while others were unstable.

### Gene structure of the *SlHsp20* genes

Structure and phases of introns/exons were determined by alignment of genomic DNA and full-length cDNA sequences of *SlHsp20* genes (Supplementary Figure S1). This information was available on Sol Genomics Network (Supplementary Table S1). It was found that among the total 42 *SlHsp20* genes, 13(30.95%) were noted to be intronless, while 22 genes (52.38%) had one intron, and only 7(16.67%) had two or more introns. Based on the number of introns, we divided the genes into three patterns (pattern one has no intron, pattern two has one intron, and pattern three has more than one) (Ouyang et al., 2009). It was evident that most *SlHsp20* genes belonged to pattern 1 and 2. Additionally, a relatively short intron length was found in SlHsp20 proteins in tomato, which was similar to the results reported on sHsp20 proteins in rice (Sarkar et al., 2009).

Using the MEME tool, 10 putative conserved motifs (motif 1 to motif 10) in *SlHsp20* gene family were identified (Supplementary Table S2). The lengths of these conserved motifs varied from 15 to 57 aa (*P* < 0.0001). Details of all the putative motifs are outlined in Table 2. Among the 10 motifs, motif 2 appeared in all putative *SlHsp20* genes, except for *SlHsp15.5*. Based on the analysis of Pfam, the full sequences of motif 1, 2, 3, 4, 5, and 8 were corresponding to the region of conserved ACD. We also found that motif 1 was in the C-terminal regions, while motif 6 appeared in the N-terminal regions generally. In addition, motif 9 was also distributed in the C-terminal regions of several genes. Notably, four *SlHsp20* genes (*SlHsp11.9, SlHsp17.3A, SlHsp14.5*, and *SlHsp15.8*) present similar patterns of motif distribution. Also, the same scenario was found in other four *SlHsp20* genes (*SlHsp17.6C, SlHsp17.7A, SlHsp17.6B*, and *SlHsp17.6A*) on chromosome 6. These results suggest that these genes may have relatively high conservation. Nevertheless, the functions of these highly conserved amino acid motifs still remain elusive.

**Table 2:** **List of the putative motifs of SlHsp20 proteins**.

**Motif**	**Width**	**Sequence**
1	41	WHRMERSCGKFMRRFRLPENANMDQIKASMENGVLTVTVPK
2	15	DLPGYKKEDIKVQVE
3	57	GRLVITGQPHQLDNFWGVTSFKKVVTLPARIDQLRTNAILTFHGCLHVHVPFAQQNL
4	21	CAFANTRIDWKETPEAHVFKV
5	29	WCRFQKDFQLPDNCNMDKISAKFENGILY
6	15	MDRVLRISGERNVEE
7	57	TPVKPTAQQPKPQHAHKDQDSTRNETMGSAESSNTQKGDNFPPRTTYPTTQAAPRKP
8	29	DVQVVDVGPPADWVKINVRATNDSFEVYA
9	21	YEDFVPTSEWVQEQDADYLLI
10	21	FDPFSIDVFDPFRELGFPGTN

**Figure 1:**
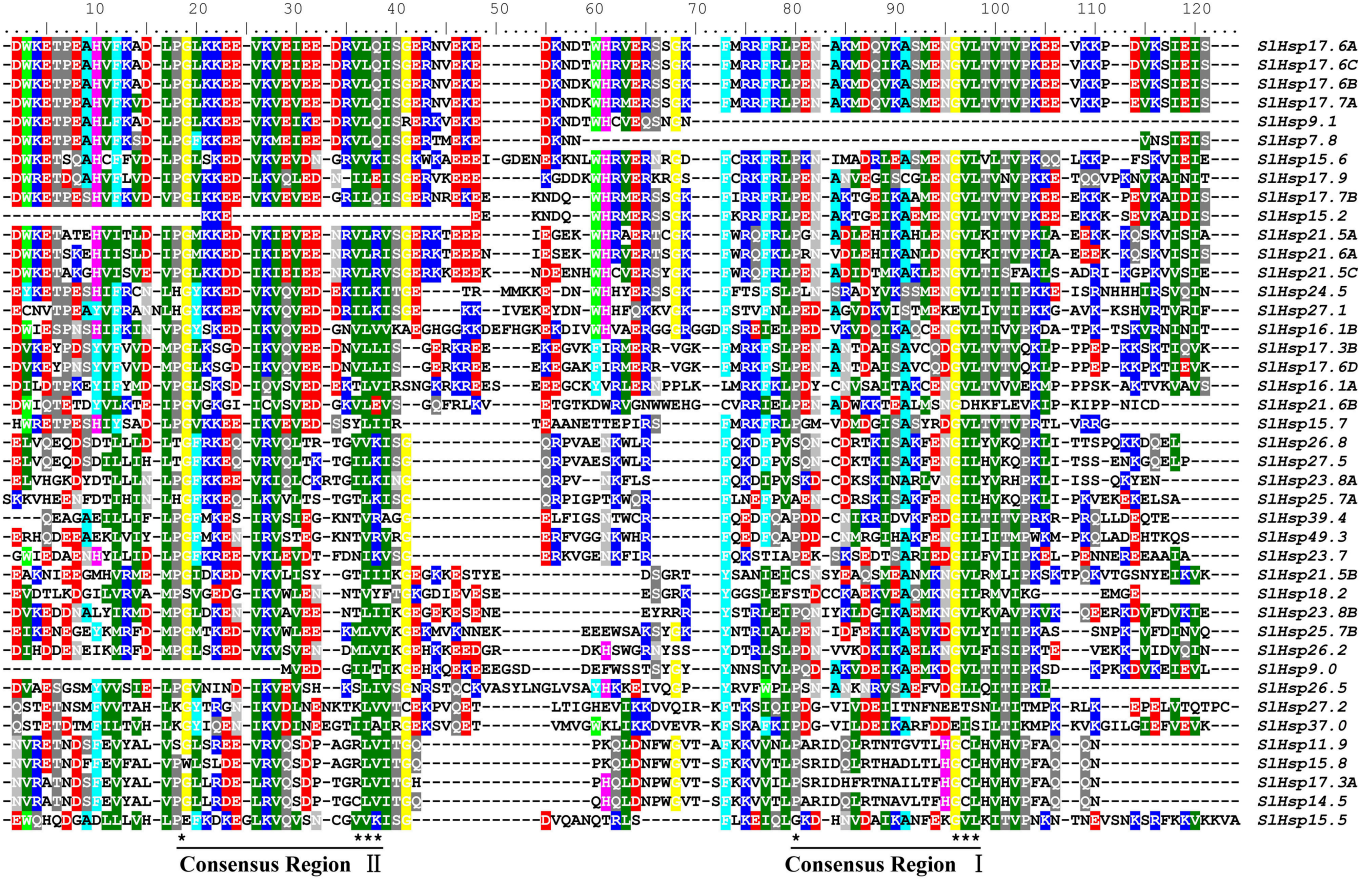
**Multiple sequence alignment of crystallin domain of SlHsp20 proteins**. Names of all the 42 members are listed on the right side of the figure. Conserved amino acid residues are indicated by color shading. Two consensus regions (consensus I and consensus II) are underlined at the bottom and the typical amino acid residues within these regions are indicated by asterisks.

It was reported that highly conserved ACD domain might be associated with the formation of multimeric complexes that are crucial for the chaperone activity of *Hsp20* genes (Waters et al., 1996; Sun et al., 2002). In the current paper, in line with a previous report (Waters et al., 1996), the highly conserved ACD in SlHsp20 proteins was divided into two parts (consensus I and consensus II) based on multiple sequence alignment (Figure 1). These two conserved regions were separated by a hydrophilic domain with variable length and characterized by residue Pro-X (14)-Gly-Val-Leu and Pro-X (14)-X-Val/Leu/Ile-Val/Leu/Ile, respectively (Caspers et al., 1995). Intriguingly, consensus I matched well with the motif 1 located on carboxyl terminal presumed by MEME.

### Phylogenetic analysis of *SlHsp20* gene family

**Figure 2:**
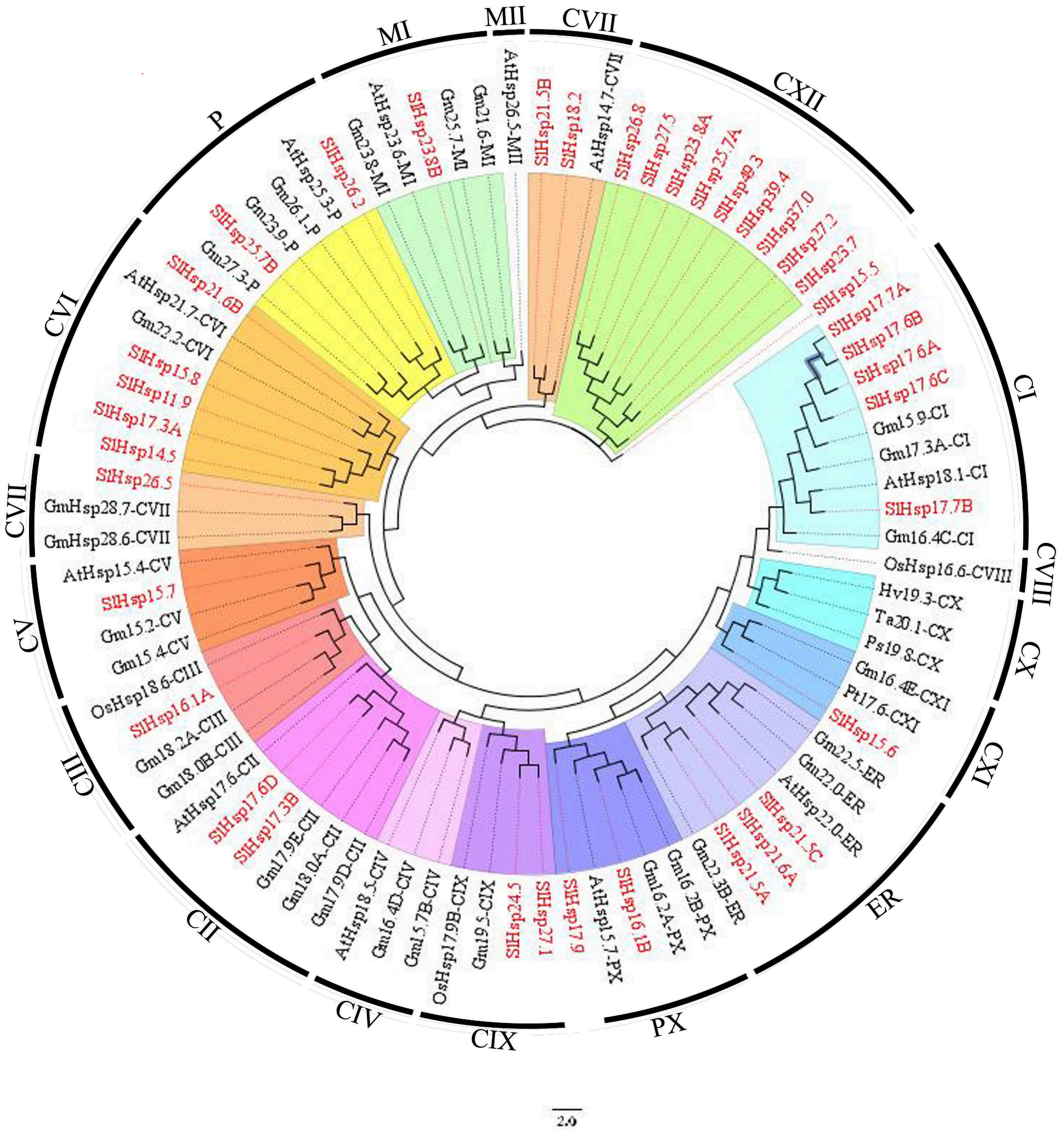
**Phylogenetic relationship of Hsp20s of tomato with diverse plant species**. The phylogenetic tree of Hsp20 proteins was constructed by Neighbor-Joining method using MEGA 7.0 software from the following species: Sl, *Solanum lycopersicum*; At, *Arabidopsis thaliana*; Gm, *Glycine max*; Os, *Oryza sativa*; Ps, *Pseudoroegneria spicata*; Hv, *Hordeum vulgare*; Ta, *Triticum aestivum*; Pt, *Populus tremula*. The putative *Hsp20* genes were divided into 17 subfamilies based on their *in silico* prediction of subcellular localization. The *SlHsp20* genes were highlighted in red. C, cytoplasmic/nuclear; ER, endoplasmic reticulum; P, plastid; Px, peroxisome; M, mitochondria.

An unrooted N-J phylogenetic tree was constructed from a complete alignment of amino acid sequences of Hsp20 proteins in tomato and other seven plant species (Figure 2). All these Hsp20 proteins were grouped into 17 distinct subfamilies. However, the SlHsp20 proteins were distributed into 13 out of 17 subfamilies, including previously identified subfamilies like CI, CII, CIII, P, Px, ER, and MI (Waters et al., 1996), recently defined CV, CVI, CVII, CIX, and CXI subfamilies (Siddique et al., 2008; Sarkar et al., 2009) and a new subfamily, CXII, identified by *in silico* prediction of subcellular localization (Supplementary Table S3). Besides, we also found two orphan *SlHsp20* genes in tomato that lack homologs genes in all seven organisms. The *SlHsp20* genes from 13 subfamilies were distributed to a variety of cellular organelles: 29 were nucleo-cytoplasmic (C) *SlHsp20* genes (9 subfamilies), 3 were endoplasmic reticulum (ER) genes, 2 were plastidial (P) genes, and 1 each for mitochondrial (M) and peroxisomal (Px) genes.

Notably, all three *SlHsp20* genes belonging to ER subfamily have a signal peptide in the N-terminal region, which was consistent with the result showed by Lopes-Caitar et al. (2013) (Table 1). The signal peptides were reported to play a positive role in facilitating the process of protein synthesis via guiding the proteins into rough endoplasmic reticulum (Bauvois, 2012). Moreover, eight *SlHsp20* genes, one from CVI subfamily and seven from CXII, had a transmembrane domain in the C-terminal region (Table 1).

We also found a close relationship between intron pattern and phylogenetic classification (Supplementary Figure S1). The result showed that the CI, CII, and ER subfamilies lacked introns. All the CVII members had more than one intron, indicating a particular phylogenetic status (Ouyang et al., 2009). The CIII, CV, CVI, CXI, MI, and Px subfamilies, together with most members of the CXII subfamily, had one intron, which may indicate a close phylogenetic relationship. In addition, gene structure may provide clues for evolutionary relationship of SlHsp20 family.

### Chromosomal localization and gene duplication

**Figure 3:**
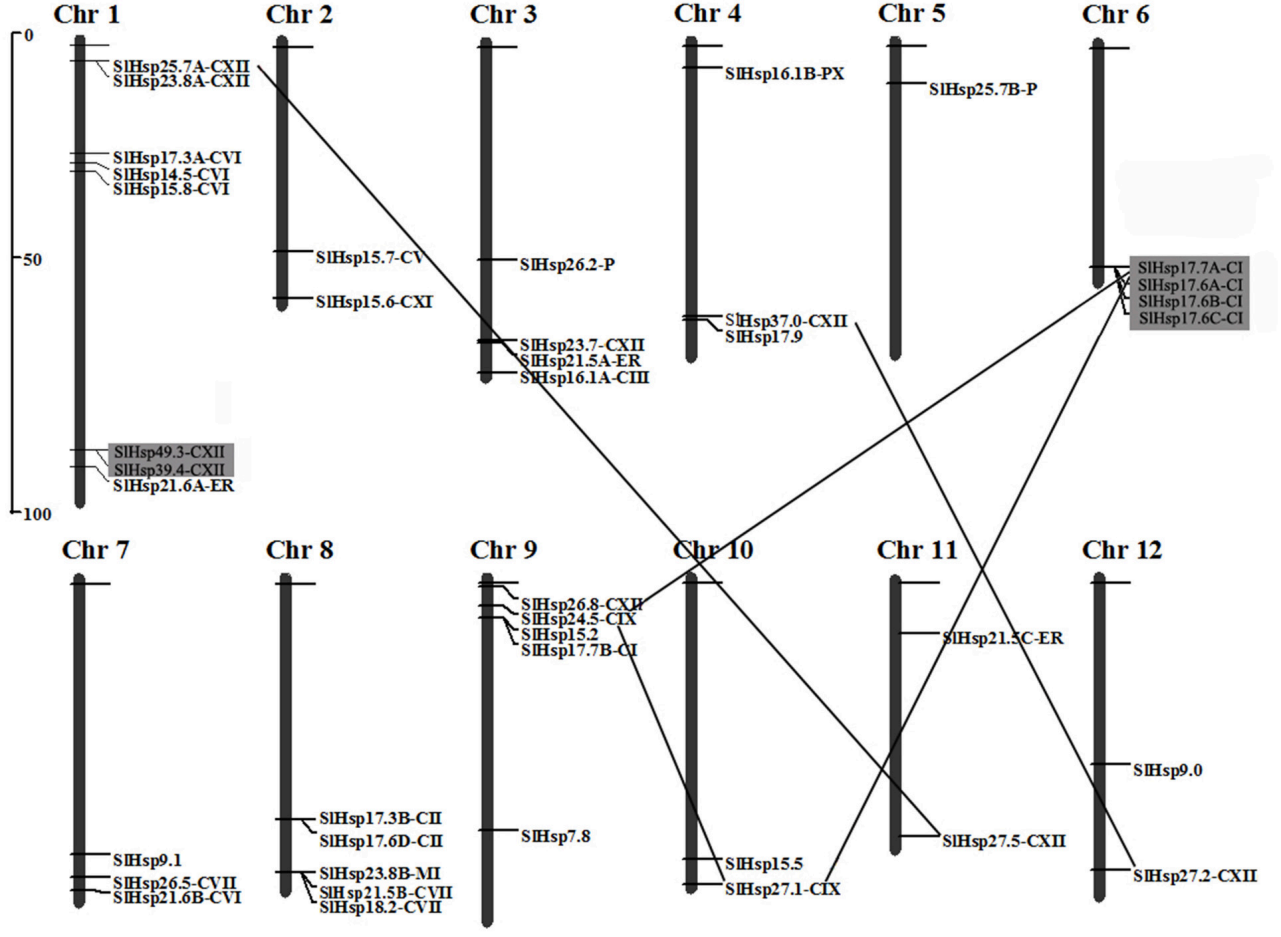
**Location and duplications of paralogous ***SlHsp20*** gene candidates on tomato chromosomes**. Chromosome numbers are shown at the top of each bar. Predicted tandem duplicated genes are indicated by gray rectangles. The *SlHsp20* genes present on duplicated chromosomal segments are connected by black lines. The scale presented on the left indicates chromosome sizes in megabases (Mb).

Out of the 42 predicted *SlHsp20* genes, 41 are randomly distributed across the 12 tomato chromosomes, except for the *SlHsp11.9* (Figure 3). Majority of the *SlHsp20* genes were located on the distal ends of the chromosomes and mainly on the lower arms. A maximum number of eight predicted *SlHsp20* genes scattered in three clusters, were present on chromosome 1.

We further performed chromosome mapping to determine the gene duplication of *SlHsp20* genes on the 12 tomato chromosomes. As shown in Figure 3, two groups of *SlHsp20* genes (*SlHsp49.3*/*SlHsp39.4* and *SlHsp17.7A*/*SlHsp17.6A*/*SlHsp17.6B*/*SlHsp17.6C*) can be identified as tandem duplication genes (Supplementary Table S4). One group (*SlHsp49.3* and *SlHsp39.4*) was from the CXII subfamily and located on chromosome 1. The other (*SlHsp17.7A, SlHsp17.6A, SlHsp17.6B*, and *SlHsp17.6C*) was from one branch of CI subfamily and juxtaposed compactly on chromosome 6, implying that the high density of *SlHsp20* genes on this chromosome was mainly due to the tandem duplication events. The chromosome location of tandemed genes showed that these four pairs of *SlHsp20* genes (*SlHsp49.3*/*SlHsp39.4, SlHsp17.7A*/*SlHsp17.6A, SlHsp17.6A*/*SlHsp17.6B, SlHsp17.6B*/ *SlHsp17.6C*) was intervened by no more than one gene and was close in distance on the chromosomes (approximately 4.6, 3.8, 8.2, and 3.9 kb) (Supplementary Table S5).

On the other hand, three segmental duplication groups were found to scatter in seven chromosomes (Figure 3; Supplementary Table S6). A duplicated segment of the *SlHsp37.0* region on the distal part of chromosome 4 was present on the same location of chromosome 12, where *SlHsp27.2* was located. *SlHsp25.7A* on chromosome 1 also showed synteny to *SlHsp27.5* localized on a duplicated segment of chromosome 11. In addition, three *SlHsp20* genes (*SlHsp17.7A, SlHsp24.5*, and *SlHsp27.1*) regions showed segmental duplication and were present on chromosome 6, 9, and 10, respectively.

### Genome-wide expression analysis of *SlHsp20* genes

**Figure 4:**
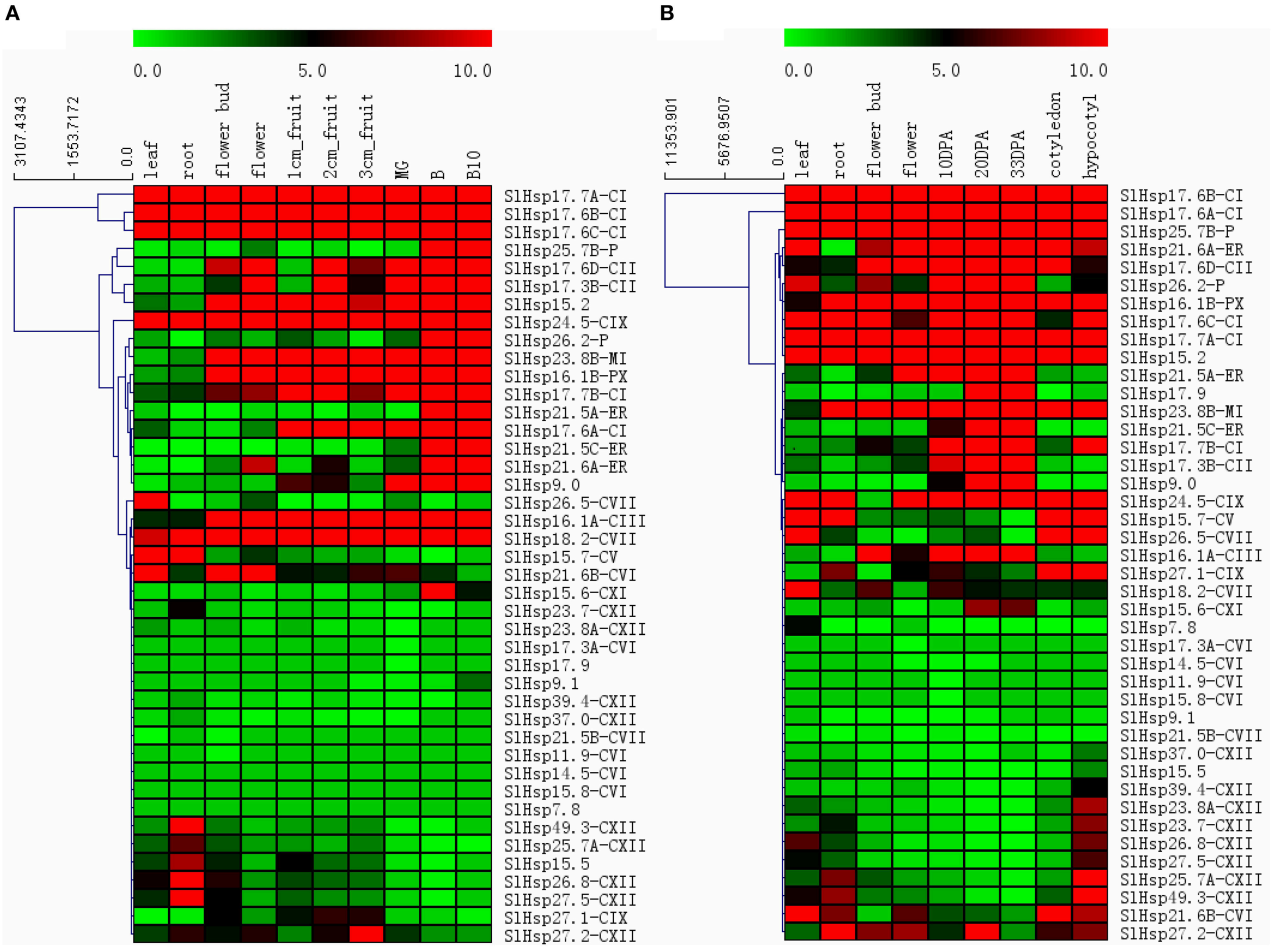
**Heat map of the expression profiles of ***SlHsp20*** genes in cultivated tomato cultivar Heniz (A) and the wild species ***S. pimpinellifolium*** (B). (A)** MG-mature green fruit; B-breaker fruit; B10-breaker+10 fruit. **(B)** DPA-days post anthesis. Cluster dendrogram is shown on the left side of heat map. Heat maps are presented in green/black/red colors that represent low/medium/high expression, respectively.

*In silico* expression patterns of the putative *SlHsp20* genes at different tissues and development stages of tomato cultivar Heinz and the wild species *S. pimpinellifolium* were analyzed (Figure 4). It showed that most of the genes (83.3%) were expressed in at least one stage (tissue) from at least one genotype. Fourteen genes (*SlHsp17.7A, SlHsp17.6B, SlHsp17.6C, SlHsp24.5, SlHsp18.2, SlHsp16.1A, SlHsp17.7B, SlHsp17.6A, SlHsp25.7B, SlHsp15.2, SlHsp21.6A, SlHsp17.6D, SlHsp16.1B, SlHsp23.8B*) were expressed constitutively in all the stages analyzed, whereas the transcripts of 12 genes (*SlHsp23.8A, SlHsp17.3A, SlHsp17.9, SlHsp9.1, SlHsp39.4, SlHsp37.0, SlHsp21.5B, SlHsp11.9, SlHsp14.5, SlHsp15.8, SlHsp7.8*, and *SlHsp15.5*) were at almost undetectable levels. Among these genes, *SlHsp17.6B* had the highest expression level in the 30 DPA fruit.

When the expression patterns of *SlHsp20* genes in vegetative organs (leaf and root) and reproductive organs (flower bud and flower) were compared between the 2 tomato genotype, 17 genes were either highly-induced (3) or barely expressed (14) (Figure 4). Conversely, two genes (*SlHsp25.7B* and *SlHsp17.6A*) displayed significantly differential expression in the various genotypes. Further, seven genes exhibited varied expression in vegetative and reproductive organs of tomato cultivar Heinz, while only two of them expressed differentially in *S. pimpinellifolium*. Notably, expression of seven genes was restricted to the leaf (*SlHsp26.5*) and root (*SlHsp23.7, SlHsp49.3, SlHsp25.7, SlHsp26.8, SlHsp27.5*, and *SlHsp15.5*) in Heniz, and only one gene was noted to root (*SlHsp7.8*) in *S. pimpinellifolium*. It indicates that these genes are regulated in a tissue-specific manner.

Expression levels of *SlHsp20* genes at breaker stage fruits were higher than that in other development stages in both genotypes. In tomato cultivar Heniz, expression of several *SlHsp20* genes (*SlHsp25.7B, SlHsp26.2, SlHsp21.5A, SlHsp21.5C*, and *SlHsp15.6*) was not almost detected in young tomato fruits (1 cm-, 2 cm-, 3 cm-, and MG fruit), but a distinct expression was observed in the breaker fruits (Figure 4A). In *S. pimpinellifolium*, two *SlHsp20* genes, *SlHsp17.9*, and *SlHsp15.6*, were only expressed in 20 DPA and 33 DPA fruits (Figure 4B). Furthermore, expression of three *SlHsp20* genes (*SlHsp23.8A, SlHsp23.7*, and *SlHsp39.4*) derived from CXII subfamily showed tissue specificity in hypocotyl. Besides, by analyzing the expression profiles of tandem and segmental duplication of *SlHsp20* genes in two genotypes, we found that two groups of tandemly *SlHsp20* genes (*SlHsp49.3*/ *SlHsp39.4*; *SlHsp17.7A*/*SlHsp17.6A*/*SlHsp17.6B*/*SlHsp17.6C*) displayed a more similar expression pattern, while difference in expression was observed for *SlHsp20* genes (*SlHsp27.1*/*SlHsp17.7A*/*SlHsp24.5*; *SlHsp37.0*/*SlHsp27.2*) in segmental duplication regions.

### Expression profiles of *SlHsp20* genes induced by different biotic and abiotic stresses

**Figure 5:**
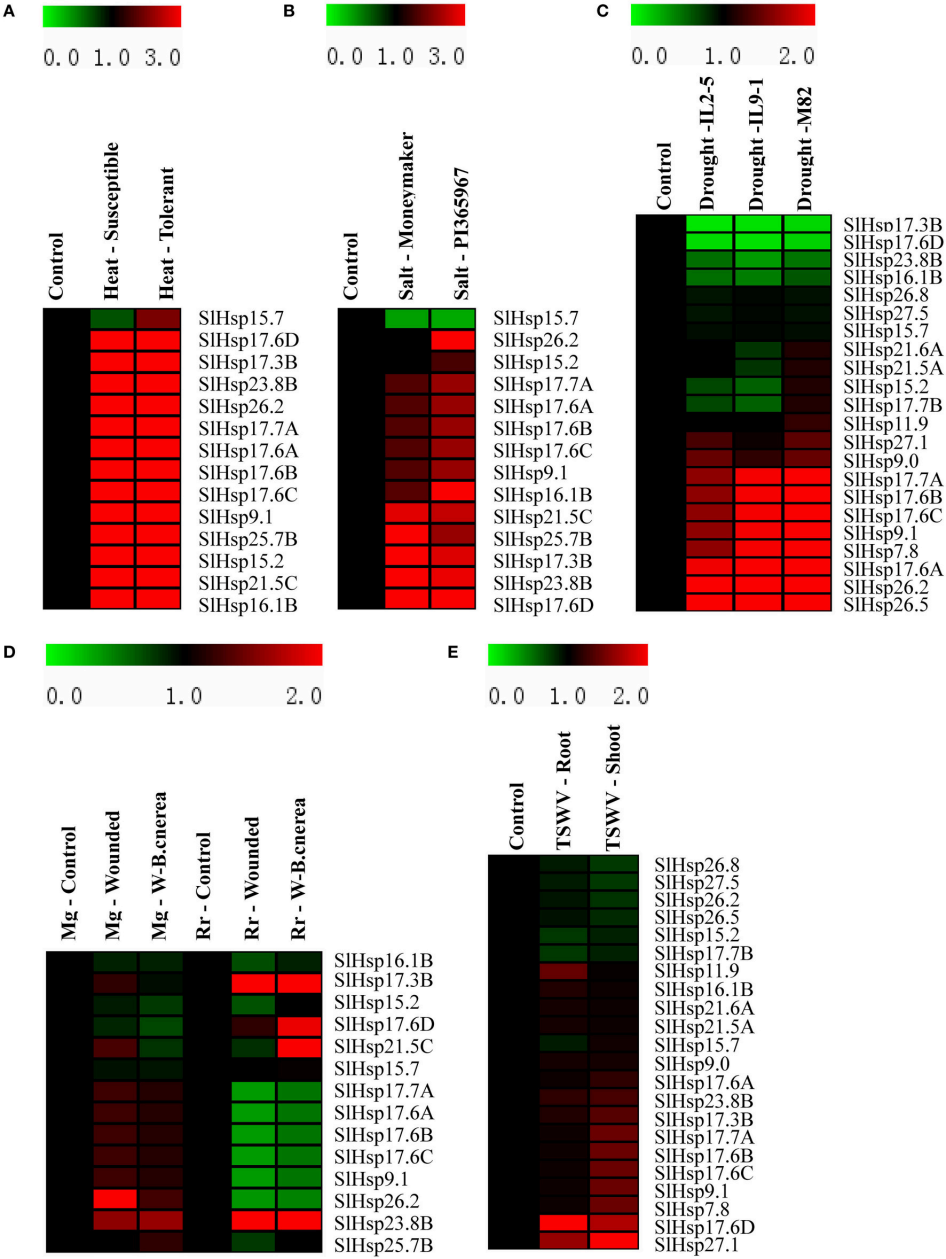
**Expression profiles of ***SlHsp20*** genes under various biotic and abiotic stresses conditions**. Blocks with colors represent decreased (green) or increased (red) transcript levels relative to the respective control. **(A)** Expression profiles of *SlHsp20* genes in tolerant and susceptible tomatoes under heat stress condition. **(B)** Expression profiles of *SlHsp20* genes under salt stress in a wild tomato genotype “PI365967” (salt-tolerant) and cultivated tomato var. moneymaker (salt-sensitive). **(C)** Expression profiles of *SlHsp20* genes under drought stress condition in two drought-tolerant lines (IL2-5 and IL9-1) and a drought-sensitive cultivar (M82). **(D)** Expression profiles of *SlHsp20* genes infected by wound and wound-inoculated with *Botrytis cinerea* in mature green (Mg) and red fruits (Rr). **(E)** Expression profiles of *SlHsp20* genes in tomato leaves and roots infected by tomato spotted wilt virus (TSWV).

To further explore the expression profiles of *SlHsp20* genes under various abiotic and biotic stresses, microarray analysis were performed (Figure 5). In the present paper, five tomato microarray data sets, belonging to two array platforms (TOM2 oligo array and Affymetrix genome array), were obtained from Tomato Functional Genomics Database (TFGD, http://ted.bti.cornell.edu/). A total of 24 probes (57%) corresponding to *SlHsp20* genes were identified, while five probes (LE17D07, LE26P10, LE26F10, LE13N06, Les.4004.1.S1_a_at) showed cross-reactivity with 12 *SlHsp20* genes (Supplementary Table S7).

Microarray-based expression analysis of tomato under various abiotic stresses revealed that expression of most of the *SlHsp20* genes were highly variable (Figures 5A–C). Expression of 13 of all tested *SlHsp20* genes, especially *SlHsp25.7B, SlHsp15.2, SlHsp21.5C*, and *SlHsp16.1B*, was drastically enhanced in resistant and susceptible tomato plants under high temperature condition, except for *SlHsp15.7* that was down-regulated in susceptible plants in response to heat stress (Figure 5A). Half of the analyzed *SlHsp20* genes in susceptible plants showed a higher expression level than those in tolerant plants. Under salt treatment condition, 12 *SlHsp20* genes displayed highly elevated expression, whereas *SlHsp15.7* was shown to be significantly down-regulated (Figure 5B). PI365967, a more salt-tolerant tomato genotype, showed relatively more responsive genes compared to tomato cultivar Moneymaker. Under drought stress condition, transcript levels of 10 and 4 *SlHsp20* genes were up-regulated and down-regulated in drought-tolerant tomato lines (IL2-5 and IL9-1) and drought-sensitive cultivar M82, respectively (Figure 5C). Among these genes, *SlHsp26.5* showed a drastic enhancement of the transcript level (more than 16 fold) in three tested tomato genotypes. The expression of *SlHsp26.2* was dramatically up-regulated in M82 (a drought-sensitive cultivar), which was much higher (four times) than that in IL2-5 and IL9-1.

Invoked by wound and the invasion of *B. cinerea*, transcript levels of three genes (*SlHsp15.7, SlHsp23.8B*, and *SlHsp16.1B*) were remained unaltered, up- and down-regulated levels, respectively, in all tested samples (Figure 5D). In mature green fruit, most of the *SlHsp20* genes displayed a stronger expression in wounded fruits than that in fruits of wound-inoculated with *B. cinerea*. In red ripe fruit, however, the expression patterns of *SlHsp20* genes showed a reverse pattern between the wounded and wound-inoculated with *B. cinerea* fruits. Interestingly, seven genes (*SlHsp17.6D, SlHsp17.7A, SlHsp17.6A, SlHsp17.6B, SlHsp17.6C, SlHsp9.1*, and *SlHsp26.2*) displayed differential expression between mature green- and red ripe fruits. In addition, under TSWV infection condition, three *SlHsp20* genes (*SlHsp11.9, SlHsp17.6D*, and *SlHsp27.1*) were up-regulated in tomato roots, three genes (*SlHsp15.2, SlHsp17.7B*, and *SlHsp15.7*) were down-regulated, and expression of the remaining genes remained unchanged (Figure 5E). In shoots of tomato, almost half *SlHsp20* genes were enhanced, six genes were reduced and expression levels of the remaining six genes were unaltered.

## Discussion

### Identification and phylogentic relationship of *SlHsp20* gene family

Using *in silico* methods to search for *Hsp20* genes in the *S. lycopersicum* genome, at least 42 putative *SlHsp20* genes were identified (Table 1). Among them, four predicted *SlHsp20* genes were excluded in the phylogenetic tree construction due to incomplete Hsp20 domain. Previously, *Hsp20* gene family in *Arabidopsis* was categorized into 12 subfamilies (CI, CII, CIII, CIV, CV, CVI, CVII, MI, MII, P, ER, and Px) (Scharf et al., 2001; Siddique et al., 2008). Likewise, four novel nucleocytoplasmic subfamilies (CVIII, CIX, CX, and CXI) were also reported on *OsHsp20* genes in rice (Sarkar et al., 2009). In the present paper, to reveal phylogenetic relationship of *Hsp20* genes, *Hsp20* genes from seven plant species, together with *Hsp20* genes from tomato, were used to construct phylogenetic tree (Figure 2). The results showed that *Hsp20* genes from the tested plant species could be grouped into 17 subfamilies, according to the method described previously (Scharf et al., 2001; Siddique et al., 2008; Sarkar et al., 2009). The *Hsp20* genes from tomato were grouped into 13 out of 17 subfamilies. A novel subfamily, CXII, was identified using *in silico* localization prediction, which was composed of 9 *Hsp20* genes from tomato (Supplementary Table S3). No SlHsp20 genes were grouped into the remaining four subfamilies (CIV, CVIII, CX, and MII subfamilies). Previous study showed that CIV and MII subfamilies may play an important role in response to various stress conditions and were developmentally regulated (Siddique et al., 2008). Members of CVIII subfamily could be heat-induced and CX subfamily might be involved in specific housekeeping functions under normal growth conditions (Sarkar et al., 2009). Similarly, several subfamilies of the Hsp20 genes in pepper also lacks, including CIV, CV, CVIII, CIX, CX, and CXI subfamilies. (Guo et al., 2015). Furthermore, the CIV and CVII subfamilies in rice Hsp20 family were absent (Sarkar et al., 2009). Thus, we were tempting to speculate that gene gain and gene loss events were occurred widely in plant species. The lack of subfamilies exists not only in tomato, but also in other species. It also revealed a wide range of genetic diversity in dicotyledons and monocotyledons.

Furthermore, we also found that most of *Hsp20* genes (69.0%) were clustered into nine nucleocytoplasmic subfamilies, which also had been described in *Arabidopsis* and rice (Scharf et al., 2001; Sarkar et al., 2009). Among these subfamilies, CXII was the largest subfamily with nine members. Therefore, we speculated that cytoplasm, as a site mainly for proteins to be synthesized, could be the primary place for *Hsp20* interacting with denatured proteins to avoid them from inappropriate aggregation and degradation (Lopes-Caitar et al., 2013). In addition, most subfamilies included *Hsp20* genes from multiple plant species and formed a mixture of groups, suggested that diversification of *SlHsp20* subfamilies predated the divergence of these species from the ancestral species.

### Organization of *SlHsp20* genes

It was reported that gene organization played a vital role in the evolution of multigene family (Xu et al., 2012). Our results showed that *SlHsp20* genes of CI, CII, and ER subfamilies had no intron (pattern one) (Supplementary Figure S1). Members of CIII, CV, CIX, MI, and PX subfamilies as well as most members of CXII subfamily had one intron (pattern two). Furthermore, these *SlHsp20* genes within the same subfamily shared a similar motif arrangement (Supplementary Table S2). This correlation between motif arrangement, intron numbers and phylogeny can be served as an additional support to their classification.

The results also showed that most of *SlHsp20* genes had no intron or one intron and the length of introns of *SlHsp20* genes with one intron is relatively short. This is concurrent with the result reported previously by the researcher that plants were prone to retain more genes with no intron or a short intron (Mattick and Gagen, 2001). In addition, the instability index of most proteins was equal or greater than 40, signifying that SlHsp20 proteins can be identified as unstable proteins (Table 1). The instability is also considered as a common trait of stress-activated proteins and thus sheds a brilliant light on the rapid induction of the *Hsp20* genes (Rao et al., 2008).

### Evolution of the *SlHsp20* gene family

Previous research reported that Hsp20 proteins were ubiquitous from single-celled creatures including bacteria to higher organisms like human (Kim et al., 1998; Waters et al., 2008; Li et al., 2009). It implied that Hsp20 proteins may have evolved in early stages of the life's history which predated to the divergence of the three domains of life (Eukarya, Bacteria, and Archaea). However, the formation of individual Hsp20 subfamilies was various. There were no Hsp20 subfamilies but only cytosolic sHsps in the green algae and then CI, CII, and P subfamilies appeared in mosses (Waters and Vierling, 1999a,b). The process generated gene families attribute to gene duplication, gene loss, and recombination (including gene conversion) (Nei and Rooney, 2005; Flagel and Wendel, 2009). The sHsps might had undergone duplication events early in the history of land plant (Waters, 2013).

Gene duplication was reported as one of the primary forces that drive the evolution processes of genetic systems and genomes (Moore and Purugganan, 2003). In this paper, chromosome mapping showed that 41 *SlHsp20* genes were located unevenly on 12 tomato chromosomes (Figure 3). The localization of most of the *SlHsp20* genes was on the terminal regions of the chromosomes, which might contribute to the occurrence of duplication events in tomato *Hsp20* gene family.

In our study, a total of 12 *SlHsp20* genes were demonstrated to be involved in gene duplication, including tandem duplication and segmental duplication (Figure 3). Two tandem duplication events (*SlHsp49.3*/*SlHsp39.4* and *SlHsp17.7A*/*SlHsp17.6A*/*SlHsp17.6B*/ *SlHsp17.6C*) and three segmental duplication events (*SlHsp25.7A*/*SlHsp27.5, SlHsp17.7A*/ *SlHsp24.5*/*SlHsp27.1*, and *SlHsp37.0*/*SlHsp27.2*) were observed in CI, CIX, and CXII subfamilies. Intriguingly, *SlHsp17.7A* on chromosome 6 was shown to participate in both tandem and segmental events. It shared a duplicated region with *SlHsp27.1* at the similar position on chromosome 10 and *SlHsp24.5* on the upper arm of chromosome 9, while *SlHsp27.1* and *SlHsp24.5* had no tandem duplicated genes in their surrounding region. Comparative analysis revealed that sequence similarity between *SlHsp17.7A* and *SlHsp27.1* was lower than that between *SlHsp17.7A* and *SlHsp17.6A*, suggesting that segmental duplication event predated the tandem duplication event in the *SlHsp17.7A* cluster, and the tandemly organized genes close to *SlHsp27.1* might be lost after the segmental duplication event (Supplementary Tables S5, S6). Similar scenario was observed for *SlHsp17.7A* and *SlHsp24.5*. Together, these results indicated that both tandem duplication and segmental duplication made significant contributions to the expansion of the *SlHsp20* gene family in tomato.

### Expression patterns of *SlHsp20* genes in different tissues

Based on RNA-Seq atlas, a spatio-temporal regulation of *SlHsp20* gene family was observed in various tissues and development stages. Under normal growth conditions, a high or preferential expression of 11 *SlHsp20* genes was found, which showed tissue- and development-specific expression in leaf, root, hypocotyl, and breaker fruit (Figure 4). All these tissue- and development-preferential expressed genes may play a critical role in growth and development of tomato and their functions still deserve further investigation. In addition, the expression behavior of some *SlHsp20* genes differed in various tissues and development stages, indicating that the SlHsp20 proteins may play diverse functional roles. We also found that four genes (*SlHsp17.7A, SlHsp17.6B, SlHsp17.6C*, and *SlHsp24.5*) were highly expressed in all the investigated tissues, implying that they might be implicated in specific housekeeping activity of tomato cell under normal growth conditions. In vegetative and reproductive organs, two *SlHsp20* genes (*SlHsp25.7B* and *SlHsp17.6A*) displayed differential expression levels between cultivated tomato and the wild relative *S. pimpinellifolium*, which indicated that inter-species divergence of gene expression was occurred and it might lead to functional specialization.

### Expression patterns of *SlHsp20* genes under abiotic and biotic stresses

Under various stress conditions, it's evident that the *SlHsp20* genes were induced to a larger extent when tomato suffered from abiotic stresses, including heat, salt, and drought treatments (Figure 5). Furthermore, we found that the heat stress inducibility of *SlHsp20* genes in susceptible plants was stronger than that in tolerant plants, which also had been demonstrated in *CaHsp20* genes (Guo et al., 2015). This finding indicated that a more efficient mechanism might have been built in the tolerant plants so that fewer *Hsp20* genes were sufficient to reduce the damage from heat shock.

Previous research had reported that the duplicated genes were easier for increasing the diversity of gene expression than single-copy genes (Gu et al., 2004). Here, we found that expression patterns of two groups of the tandemly duplicated genes were highly similar, which reflected that these *SlHsp20* genes might share similar induction mechanisms and network (Ouyang et al., 2009). Actually, compared with tandem duplications, *SlHsp20* genes in segmental duplicated group showed a more differential expression behavior. For example, the different expression between *SlHsp37.0*-CXII and *SlHsp27.2*-CXII suggested that segmentally duplicated genes may also exhibit divergent expression patterns (Waters, 2013). Earlier study revealed that distantly related duplicate genes may exist functional redundancy and have more chances to acclimatize than the single-copy genes (Gu et al., 2003). Thus, the duplicated *SlHsp20* genes might go through crucial diversification after duplication events, which eventually caused neo-functionalization (Ouyang et al., 2009). It was also reported as a means for the retention of those duplicated genes in a genome (Force et al., 1999).

## Conclusions

In the current study, 42 putative *SlHsp20* genes were identified in tomato. Subsequently, characterization of *SlHsp20* genes was performed through integration of comprehensive sequence, genome organization and expression profile analysis among different tissues and under different stresses (heat, drought, salt, TSWV, and *B. cinerea*) by using RNA-seq and microarray atlas. This study provided a comprehensive understanding of the *SlHsp20* gene family in tomato and made a basis for working out the functional roles of the *Hsp20* genes in the Solanaceae family in the future.

## Author contributions

Conceived and designed the experiments: HW and YY. Performed the experiments: JY, YC, MR, QY, RW, ZL, GZ, and ZY. Analyzed the data: JY, YC, and KF. Wrote the paper: JY and YC. All authors have read and approved the manuscript.

### Conflict of interest statement

The authors declare that the research was conducted in the absence of any commercial or financial relationships that could be construed as a potential conflict of interest. The reviewer CY and handling Editor declared their shared affiliation, and the handling Editor states that the process nevertheless met the standards of a fair and objective review.

## Supplementary material

Supplementary Figure S1 **Intron/exon configurations of *SlHsp20* genes**. The numbers (0, 1, 2) represented introns phases, which indicated three different splicing patterns.

Supplementary Table S1 **Genomic sequences, coding sequences and amino acid sequences of *SlHsp20* genes**.

Supplementary Table S2 **Organization of putative motifs in SlHsp20 proteins**. Ten putative motifs are represented by numbered color boxes. Names of all the proteins were indicated on the left side.

Supplementary Table S3 **Predicted subcellular location of *SlHsp20* genes**.

Supplementary Table S4 **Homology matrixes of predicted tandem *SlHsp20* gene sequences**.

Supplementary Table S5 **Tandemly duplicated *SlHsp20* genes**.

Supplementary Table S6 ***SlHsp20* genes localized on duplicated segments of the tomato genome**.

Supplementary Table S7 **Information of probe sets used for microarray expression analysis**.
