# Supplementary figures and images for: Genome-Wide Identification and Expression Profiling of Tomato Hsp20 Gene Family in Response to Biotic and Abiotic Stresses
Source: Front Plant Sci. 2016 Aug 17;7:1215. doi: 10.3389/fpls.2016.01215 (PMC4987377; doi:10.3389/fpls.2016.01215)

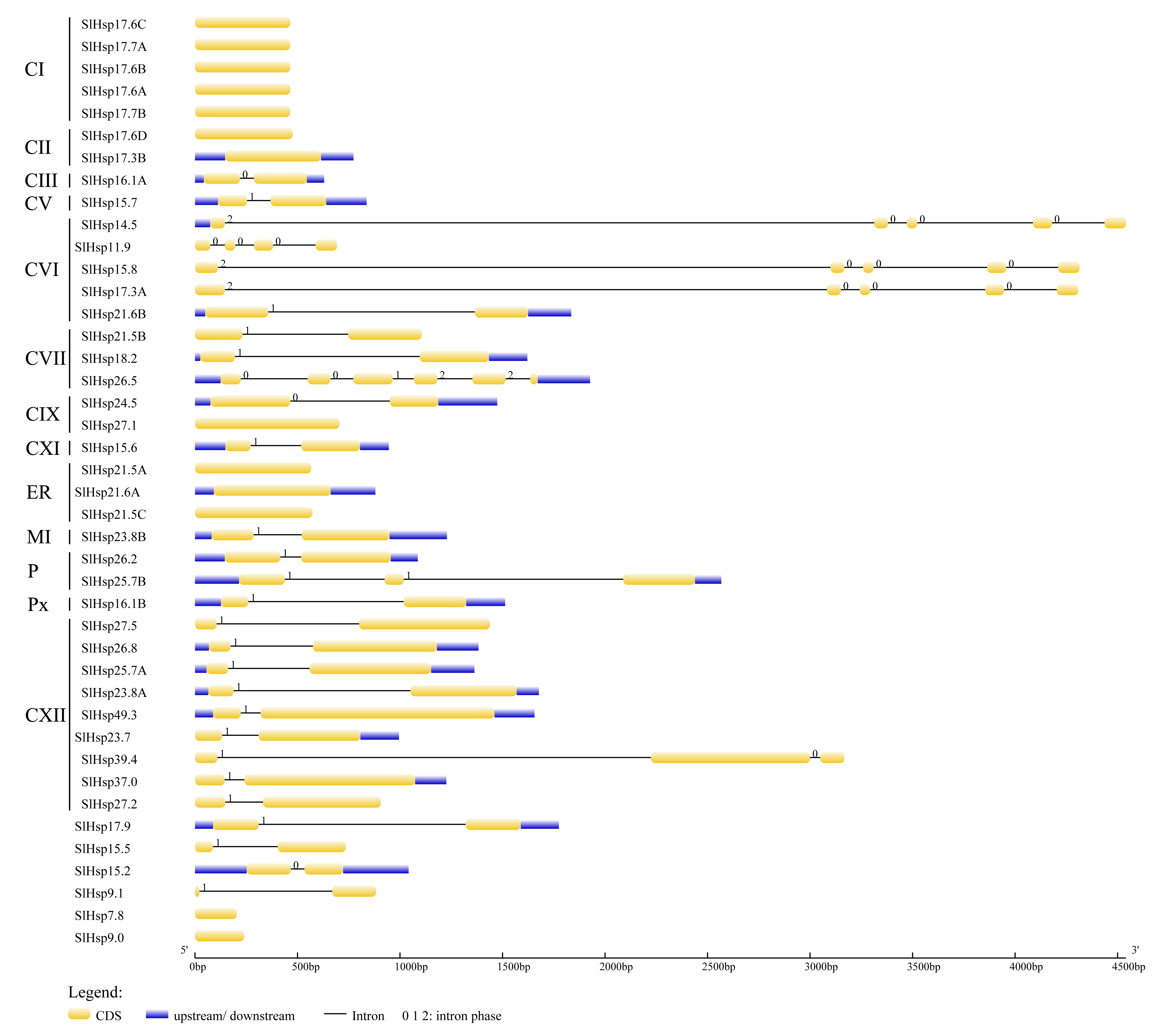

Supplement: Supplementary Figure S1 — Intron/exon configurations of SlHsp20 genes. The numbers (0, 1, 2) represented introns phases, which indicated three different splicing patterns. [file Image1.JPEG]
