# Supplementary material for: Genome-Wide Identification and Expression Profiling of Tomato Hsp20 Gene Family in Response to Biotic and Abiotic Stresses
Source: Front Plant Sci. 2016 Aug 17;7:1215. doi: 10.3389/fpls.2016.01215 (PMC4987377; doi:10.3389/fpls.2016.01215)
Supplement: Supplementary Table S4 — Homology matrixes of predicted tandem SlHsp20 gene sequences. [file Table4.DOC]

**Supplementary Table S4. Homology matrixes of predicted tandem *SlHsp20* genes sequences.**

| **Gene name** | *SlHsp49.3* | *SlHsp39.4* | *SlHsp17.7A* | *SlHsp17.6A* | *SlHsp17.6B* | *SlHsp17.6C* |
| --- | --- | --- | --- | --- | --- | --- |
| *SlHsp49.3* | 100% |  |  |  |  |  |
| *SlHsp39.4* | 81.7% | 100% |  |  |  |  |
| *SlHsp17.7A* | 37.2% | 39.4% | 100% |  |  |  |
| *SlHsp17.6A* | 37.4% | 38.6% | 91.2% | 100% |  |  |
| *SlHsp17.6B* | 37.2% | 39.4% | 98.8% | 91.9% | 100% |  |
| *SlHsp17.6C* | 35.7% | 37.7% | 91.4% | 91.9% | 91.7% | 100% |
