# Supplementary material for: Genome-Wide Identification and Expression Profiling of Tomato Hsp20 Gene Family in Response to Biotic and Abiotic Stresses
Source: Front Plant Sci. 2016 Aug 17;7:1215. doi: 10.3389/fpls.2016.01215 (PMC4987377; doi:10.3389/fpls.2016.01215)
Supplement: Supplementary Table S5 — Tandemly duplicated SlHsp20 genes. [file Table5.DOC]

**Supplementary Table S5. Tandemly duplicated *SlHsp20* genes.**

| **Group** | **Gene** | **Duplicate** | **%Identity^a^** | **%Homology** | **Genes** **intervening** | **Distance (bp)** |
| --- | --- | --- | --- | --- | --- | --- |
| I | SlHsp49.3 | SlHsp39.4 | 76.40% | 81.7% | 1 | 4619 |
| II | SlHsp17.7A | SlHsp17.6A | 91.40% | 91.2% | 1 | 3803 |
| II | SlHsp17.6A | SlHsp17.6B | 91.61% | 91.9% | 1 | 8193 |
| II | SlHsp17.6B | SlHsp17.6C | 91.83% | 91.7% | 0 | 3923 |

^a^Homology between proteins encoded by tandemly duplicated genes
