# Supplementary material for: Genome-Wide Identification and Expression Profiling of Tomato Hsp20 Gene Family in Response to Biotic and Abiotic Stresses
Source: Front Plant Sci. 2016 Aug 17;7:1215. doi: 10.3389/fpls.2016.01215 (PMC4987377; doi:10.3389/fpls.2016.01215)
Supplement: Supplementary Table S6 — SlHsp20 genes localized on duplicated segments of the tomato genome. [file Table6.DOC]

**Supplementary Table S6.** ***SlHsp20* genes localized on duplicated segments of the tomato genome.**

| **Gene1** | **Locus** | **Chr.** | **Gene2** | **Locus** | **Chr.** | **Overall identity^a^** |
| --- | --- | --- | --- | --- | --- | --- |
| SlHsp25.7A | Solyc01g009200 | 1 | SlHsp27.5 | Solyc11g071560 | 11 | 44.98% |
| Slhsp37.0 | Solyc04g071490 | 4 | Slhsp27.2 | Solyc12g056560 | 12 | 39.77% |
| SlHsp17.7A  SlHsp24.5  SlHsp27.1 | Solyc06g076520  Solyc09g011710  Solyc10g086680 | 6  9  10 | SlHsp24.5  SlHsp27.1  SlHsp17.7A | Solyc09g011710  Solyc10g086680  Solyc06g076520 | 9  10  6 | 25.83%  40.28%  24.05% |

^a^Percentage homology between proteins encoded by genes localized on duplicated segments
